# Eggplant Peels as a Valuable Source of Anthocyanins: Extraction, Thermal Stability and Biological Activities

**DOI:** 10.3390/plants10030577

**Published:** 2021-03-18

**Authors:** Nina-Nicoleta Condurache (Lazăr), Constantin Croitoru, Elena Enachi, Gabriela-Elena Bahrim, Nicoleta Stănciuc, Gabriela Râpeanu

**Affiliations:** 1 Faculty of Food Science and Engineering, Dunarea de Jos University of Galati, 111 Domneasca Street, E Building, 800201 Galati, Romania; nina.condurache@ugal.ro (N.-N.C.); elena.ionita@ugal.ro (E.E.); gabriela.bahrim@ugal.ro (G.-E.B.); nicoleta.stanciuc@ugal.ro (N.S.); 2 Academy of Agricultural and Forestry Sciences, 61 Marasti Blvd, 011464 Bucharest, Romania; c.croitoru@sodinal.com

**Keywords:** eggplant peels, bioactive compounds, anthocyanins, extraction methods, thermal degradation, biological activity

## Abstract

This study aimed to use eggplant peels as a potential source of anthocyanins with biological activities. Two different extraction methods were tested in order to obtain extracts with a high anthocyanin content. The selected methods were the solid–liquid extraction (SLE) and ultrasound-assisted extraction (UAE) methods. For each method, two concentrations of ethanol (EtOH) were used, while varying the extraction time and temperature. Based on the results, the extracts obtained by SLE using EtOH 96% after 30 min of extraction at 50 °C showed the highest anthocyanin concentration. The UAE allowed the best results with EtOH 96% after 30 min at 25 °C. Both selected extracts showed similar chromatographic profiles, with delphinidin 3-*O*-rutinoside as the major anthocyanin, but in a higher concentration in UAE. The extracts also presented inhibitory activity against lipoxygenase (LOX), lipase, and α-amylase, thus suggesting a possible involvement in reducing the risk of various disorders. The first order kinetic model was used to predict the changes that can occur in the anthocyanin content and antioxidant activity from the eggplant peel extract. The calculated kinetic and thermodynamic parameters confirm the irreversible degradation of phytochemicals.

## 1. Introduction

Eggplant fruits (*Solanum melongena* L.) are widely consumed around the world due to their generous composition of nutraceuticals [1]. In recent years, many researchers concentrated their work on the biorecovery of valuable compounds from plant by-products. [2]. The by-products that come from the processing of vegetables and fruits are rich in both primary and secondary metabolites. A special interest from the academic community and industry is given to the secondary ones [1]. These compounds comprise terpenes, alkaloids, and phenolic compounds [3]. Phenolic compounds have gained great attention due to their properties, which can make them beneficial against a variety of disorders. Metabolic syndrome (MS) is among them and refers to a series of cardio-metabolic disorders, such as obesity, insulin resistance, dyslipidemia, and hypertension. Moreover, polyphenols can be involved in reducing the risk of MS due to the high antioxidant activity they exert, which can trigger other defense mechanisms [1]. However, their ability to cross the intestinal barrier and to enter into the blood system is low. To achieve any health effect, they must be absorbed into the circulatory system and delivered to the appropriate location within the body [4].

Similar to other vegetables and fruits, eggplants contain a wide range of phenolics. For example, the eggplant pulp is the richest source of phenolic acids from all vegetables that belong to the *Solanaceae* family. The eggplant’s leaves are a good source of kaempferol. In the peels are found several types of compounds, although the most important are the anthocyanins [1]. The purple color of the eggplant peels is given by the glycosides of delphinidin [5,6].

Different in vitro and in vivo studies have shown the importance of anthocyanins. Their antioxidant activity could provide antioxidative stress [7] and anti-inflammatory effects [8], protection against UV radiation playing a key role in skin aging, antidiabetic [9,10] and antimicrobial activity [11], and anticancer and antitumor effects [12,13,14,15].

Anthocyanins are found in the outer layers of eggplants. The fact that they can bind to sugars and proteins or that each plant matrix has a different composition makes their extraction difficult [16]. The recovery of phenolic compounds from different raw materials or agro-industrial by-products can be performed by traditional extraction methods. In general, these methods are characterized by a low efficiency due to the prolonged heat treatment [5]. This could cause the degradation of the sensitive compounds and also induce energy, time, and solvent consumption, as explained by Barba et al. [17]. The so-called assisted methods, such as ultrasounds, have been recently used extensively to preserve the sensitive compounds. The main advantages provided by these techniques are high extraction efficiency, high quality of the final product at a lower temperature, and less energy/solvent consumption [17].

Despite all the benefits that anthocyanins can provide, they can degrade, easily turning into colorless or insoluble brown pigments. There are various factors affecting the anthocyanins’ stability, including temperature. Heat treatment is an industrial process applied to extend and preserve the shelf life of products and usually leads to significant losses of the phenolic compounds [18].

In the literature, solid–liquid extraction (SLE) and ultrasound-assisted extraction (UAE) methods have been used to extract the anthocyanins from many plant materials, even eggplant peels. However, there are no reported data on the comparison between the SLE and UAE of eggplant peel anthocyanins. In this regard, SLE and UAE methods were comparatively used in our study to obtain a high-quality extract, in terms of high anthocyanin content and biological activities. For each extraction method, different solvent concentrations, times, and temperatures were used. The extracts were analyzed in terms of total anthocyanin content (TAC) and antioxidant activity. Further, the selected extracts with the highest anthocyanin content were analyzed by High-Performance Liquid Chromatography (HPLC) to identify and characterize the major compounds. Additionally, the extracts were analyzed for their inhibitory activity against lipase, α-amylase, and LOX, enzymes associated with MS. A precise understanding of the kinetic and thermodynamic parameters is necessary to predict the nutritional and functional changes during industrial processing. Therefore, the extract with the highest TAC was thermally treated, and the kinetic and thermodynamic parameters were calculated.

## 2. Results and Discussion

### 2.1. The SLE Method

SLE is among the most widely used and easiest methods that consists of direct extraction of the plant material with a suitable solvent. This type of extraction involves the phenolics mass transfer from the cell walls into the extraction solvent [16]. Temperature, solvent concentration, or time could influence the extraction efficiency. To extract the anthocyanins from the eggplant peels with the SLE method, two different EtOH concentrations (70% and 96%) acidified with 1N hydrochloric acid (HCl) were used. The EtOH was chosen due to the recovery rate of the phenolic compounds, its nontoxicity, and its suitability for polar compounds. Because every matrix has a distinct composition, and phenolics have variable solubility characteristics, it is necessary to investigate the solvent concentration efficiencies. Additionally, the mixture of solvent and water can decrease solvent consumption [16]. In order to select the most suitable extraction parameters, two temperatures (25 and 50 °C) and different extraction times (30, 60, and 120 min) were tested. The extraction of anthocyanins is also affected by its duration. The extraction yield may increase as time increases, or it may cause the degradation of anthocyanins mainly due to oxidation. Additionally, high temperatures can improve the extraction efficiency by increasing the solubility, diffusion coefficients, and mass transfer rate of the compounds. However, high temperatures combined with a longer extraction time can lead to phenolic degradation [16].

Table 1 presents the extracts’ phytochemical content obtained by the parameter variation. According to our results, it could be appreciated that certain parameters had a significant effect on the anthocyanin extraction (*p* < 0.05). Although different studies have claimed that pure EtOH has a lower extraction yield compared to aqueous EtOH (50% and 70%) [5], our results showed the opposite. The highest TAC was obtained for the extraction with EtOH 96% at 50 °C (Table 1a). This could be explained by the fact that the acid strength combined with the solvent concentration and the temperature resulted in stronger cell wall breaking. The other extracts also presented high TAC. The increased temperature was positively associated with a higher TAC for both EtOH concentrations (*p* < 0.05). However, it can be noticed that there is no significant influence of the extraction time (*p* > 0.05) on TAC, regardless of the temperature and solvent concentrations. Hosseini et al. [19] tested different EtOH concentrations and reported higher TAC by the conventional extraction of anthocyanins from eggplant peels with water, EtOH, and HCl (50:48:2 ratio) than with the concentrated EtOH. Boulekbache-Makhlouf et al. [20] reported lower eggplant peel anthocyanin content after using EtOH 70% acidified with 0.2% formic acid.

Phenolic compounds from eggplants are known for their in vitro free radical scavenging activity. Table 1b presents the extracts’ antioxidant activities obtained after the SLE parameters combination. The highest antioxidant activity corresponds to the extracts obtained with EtOH 70% at 50 °C. Probably, the polarity of EtOH and water combined with the temperature facilitates the extraction of phenolics, thus providing a high antioxidant activity to the extract. However, the other extracts also presented high antioxidant activities. The extraction with EtOH 70% was significantly influenced by the temperature, with higher results being obtained at a higher temperature (*p* < 0.05). In contrast, for the EtOH 96%, the temperature presented a significant influence only after 60 min of extraction (*p* < 0.05). At 25 °C, EtOH 70% allowed significantly higher results after 60 min of extraction than 30 min (*p* < 0.05). The same behavior was observed at 50 °C with EtOH 96%. Our results are in agreement with those reported by other authors. Hosseini et al. [19] obtained a higher antioxidant activity for an eggplant peel extract after using EtOH, water, and HCl in a 50:48:2 ratio at room temperature rather than with pure EtOH.

### 2.2. The UAE Method

The UAE represents a faster alternative to conventional extraction methods. This technique is based on the energy derived from sound waves with frequencies higher than 20 kHz [21]. Table 1a presents the phytochemicals extracted with two EtOH concentrations at 25 and 50 °C and different treatment times. The highest TAC was acquired by the extraction at 25 °C with EtOH 96%. The solvent concentration significantly influenced the TAC after 30 min of extraction, EtOH 96% extracting higher contents than EtOH 70% for both temperatures (*p* < 0.05). The extracts obtained with EtOH 96% at 50 °C presented a significantly lower TAC compared to those at 25 °C (*p* < 0.05). The time did not significantly affect the extraction (*p* > 0.05). The results exhibited that the exposure for 30 min to ultrasounds is enough to extract a high content of anthocyanins. Horincar et al. [22] also reported lower TAC values from an eggplant peel extract obtained with EtOH 70% acidified with 1N HCl after 30 min at 40 °C. Dumitrascu et al. [23] reported higher TAC for higher EtOH concentrations at 50 °C after 40 min of phenolic compound extraction from cornelian cherry fruits.

As in the SLE case, using EtOH 70% led to extracts with a significantly higher antioxidant activity at 25 °C (*p* < 0.05). Thus, the highest antioxidant activity was obtained with 70% EtOH after 15 min of extraction at 25 °C (Table 1b). At 50 °C, the EtOH concentration did not significantly influence the antioxidant activity (*p* > 0.05). The temperature increase led to a significant increase in the antioxidant activity only for the extracts obtained with EtOH 96%, regardless of the time, but also for the extract with EtOH 70% after 30 min of extraction (*p* < 0.05). Time significantly influenced the antioxidant activity only for the extract with EtOH 70% at 25 °C (*p* < 0.05). Compared to our results, Horincar et al. [22] obtained lower antioxidant activity by eggplant peels ultrasound extraction using EtOH 70% acidified with 1N HCl for 30 min at 40 °C.

Both extraction methods led to the highest anthocyanin contents using EtOH 96% (Table 1a). At 25 °C, there were no significant differences between the TAC obtained with both methods after 30 min (*p* > 0.05). However, at 50 °C, SLE extracted significantly higher TAC than UAE (*p* < 0.05) after 30 min. SLE extracts obtained with EtOH 70% at 50 °C and with 96% at 25 °C presented higher antioxidant activities than those obtained by UAE (Table 1b). On the contrary, UAE extraction with EtOH 70% at 25 °C provided extracts with significantly higher antioxidant activity than SLE (*p* < 0.05).

By comparison, at the same EtOH concentration, the use of UAE brings the advantage of halving the temperature, which enables energy savings and reduces the risk of phenolic degradation. Authors such as Caldas et al. [24] reported higher phenolic compound recovery from grape skin in a shorter time with UAE than SLE.

Based on the TAC obtained, the extracts chosen for further experiments were the following: the one obtained by SLE using EtOH 96% after 30 min of extraction at 50 °C and the one obtained by UAE using EtOH 96% after 30 min at 25 °C.

### 2.3. HPLC Analysis of the Anthocyanins

**Figure 1:**
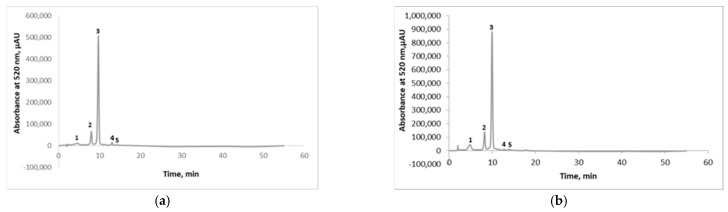
Chromatographic profile of eggplant peel extracts obtained by SLE with EtOH 96% for 30 min at 50 °C (**a**) and UAE with EtOH 96% for 30 min at 25 °C (**b**): Peak 1—delphinidin 3-*O*-rutinoside-5-glucoside; Peak 2—delphinidin 3-*O*-glucoside; Peak 3—delphinidin 3-*O*-rutinoside; Peak 4—cyanidin 3-*O*-rutinoside; Peak 5—petunidin 3-*O*-rutinoside.

In order to characterize the anthocyanin profile from the extracts obtained by the two extraction methods, the analysis of the chromatographic profile was performed by HPLC (Figure 1). 

**Figure 2:**
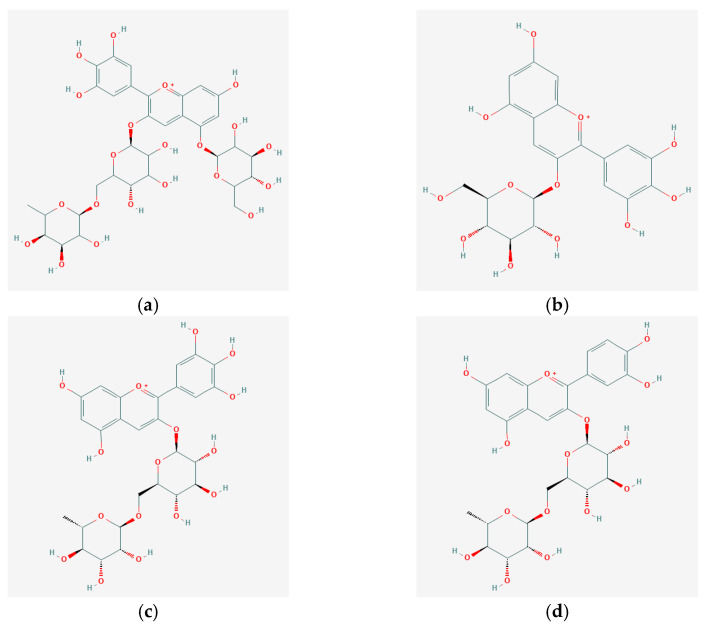

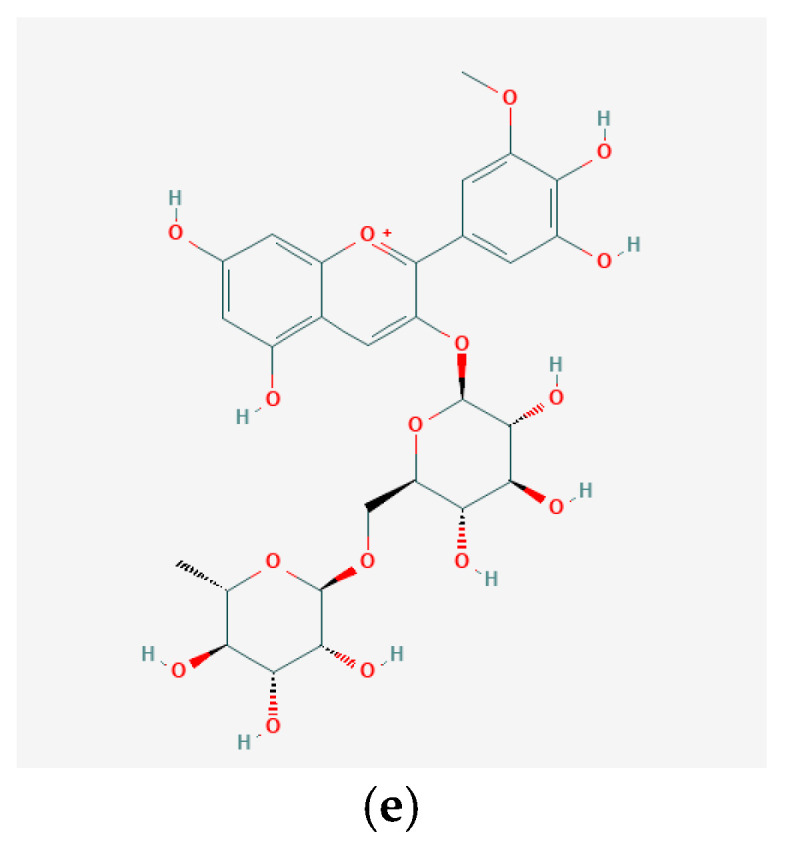
Chemical structure of the anthocyanin glycosides identified in the eggplant peel extracts: delphinidin 3-*O*-rutinoside-5-glucoside (**a**); delphinidin 3-*O*-glucoside (**b**); delphinidin 3-*O*-rutinoside (**c**); cyanidin 3-*O*-rutinoside (**d**), and petunidin 3-*O*-rutinoside (**e**). [26].

The anthocyanins from the eggplant peel extracts were identified based on the retention time and by comparison with the available standards and existing data in the literature. The identification wavelength was 520 nm. The chromatographic profile revealed the presence of five compounds: delphinidin 3-*O*-rutinoside-5-glucoside (Figure 2a), delphinidin 3-*O*-glucoside (Figure 2b), delphinidin 3-*O*-rutinoside (Figure 2c), cyanidin 3-*O*-rutinoside (Figure 2d), and petunidin 3-*O*-rutinoside (Figure 2e). Three of the compounds were assessed based on the available standards, while the other two compounds were presumptively identified based on the data reported in the scientific literature [5,25].

The SLE extract (Figure 1a) showed the following relative percentage concentration to the concentration of total anthocyanins: delphinidin 3-*O*-rutinoside-5-glucoside (9.35%), delphinidin 3-*O*-glucoside (9.72%), delphinidin 3-*O*-rutinoside (73.55%), cyanidin 3-*O*-rutinoside (1.97%), and petunidin 3-*O*-rutinoside (0.42%). The UAE extract showed the followed concentrations of anthocyanins (Figure 1b): delphinidin 3-*O*-rutinoside-5-glucoside (7.30%), delphinidin 3-*O*-glucoside (12.18%), delphinidin 3-*O*-rutinoside (75.34%), cyanidin 3-*O*-rutinoside (1.47%), and petunidin 3-*O*-rutinoside (0.54%). As such, the quantitative analysis followed the use of the standard calibration curves for the three aforementioned compounds, such as delphinidin 3-*O*-glucoside, that presented a concentration of 4 mg/100 g dw for the SLE and 5 mg/100 g dw for the UAE; delphinidin 3-*O*-rutinoside as the major anthocyanin presented a concentration of 157 mg/100 g dw for the SLE and 562 mg/100 g dw for the UAE, whereas cyanidin 3-*O*-rutinoside presented a content lower than 1 mg/100 g dw for both of the analyzed samples. Our results are in agreement with Ferarsa et al. [5] and Dranca and Oroian [25] who also identified five anthocyanins in different eggplant peel extracts, with delphinidin 3-*O*-rutinoside as the major one.

### 2.4. In Vitro Enzymes Inhibition by Eggplant Peels Extract

LOX catalyzes the formation of hydroperoxides from polyunsaturated fatty acids, which leads to several inflammation-related disorders, such as MS [27]. High levels of pancreatic lipase in the human body are responsible for pancreatic disorders and obesity due to their function in catalyzing the hydrolysis of triglycerides into fatty acids. Various researchers have demonstrated in vivo and in vitro that polyphenols can decrease and even inhibit the enzymatic activity of lipase in the human body if they are absorbed in the intestinal tract [28,29]. α-amylase is among the enzymes associated with diabetes that catalyze the hydrolysis of carbohydrates into simple sugars. A therapeutic approach that can decrease hyperglycemia is to stop the production and/or absorption of glucose by inhibiting the responsible enzymes [30]. Phenolic compounds are positively associated with reducing the risk of MS by inhibiting the responsible enzymes. Cyanidin 3-*O*-glucoside, delphinidin 3-*O*-glucoside, and petunidin 3-*O*-glucoside are known to inhibit cyclooxygenase enzyme activities related to various disorders [31]. Metformin hydrochloride is the most commonly used synthetic α-amylase inhibitor in the treatment of type 2 diabetes [32]. Orlistat is the only lipase inhibitor available on the market involved in the fight against obesity [33]. The side effects of these drugs led to research for natural alternatives with lesser side effects. These two drugs were used as positive controls in our study.

In our study, the in vitro effects of the selected eggplant peel extracts on the enzymes associated with several disorders were assessed in three enzymes (LOX, lipase, and α-amylase) and three extract concentrations. The inhibitory data are summarized in Table 2 and Table 3.

The extracts obtained by the two tested methods presented moderate enzyme inhibition activities. UAE extract showed significantly higher in vitro LOX inhibitory percentages than SLE extract for all tested concentrations (*p* < 0.05). However, the IC50 values of both extracts presented no significant differences (*p* > 0.05). When compared to the positive control, both extracts presented significantly lower in vitro LOX inhibitory percentages and IC50 values (*p* < 0.05). Both extracts showed similar in vitro lipase and α-amylase inhibitory percentages, as shown in Table 2 and Table 3. Compared with orlistat used as a standard, all extracts were found to show significantly weak lipase inhibitory activity (*p* < 0.05). However, when compared to metformin hydrochloride used as a standard for α-amylase, there were significant differences only at 0.1 mg/mL concentration (*p* < 0.05). Therefore, the eggplant peel extracts showed LOX, lipase, and α-amylase inhibition activity. This suggests that by increasing the bioavailability of anthocyanins from the eggplant peels in the human body, they might be involved in reducing glucose and lipids metabolism. Our results are in agreement with Kwon, Apostolidis, and Apostolidis [34], who reported moderate to high α-amylase inhibition capacity for different eggplant extracts. Mojica, Berhow, and Gonzalez de Mejia [35] also reported that the anthocyanins from black beans inhibit α-glucosidase and α-amylase.

Based on the HPLC chromatographic profiles obtained and biological activities, the extract chosen for further experiments was the one obtained by UAE using EtOH 96% after 30 min at 25 °C.

### 2.5. Thermal Degradation

**Figure 3:**
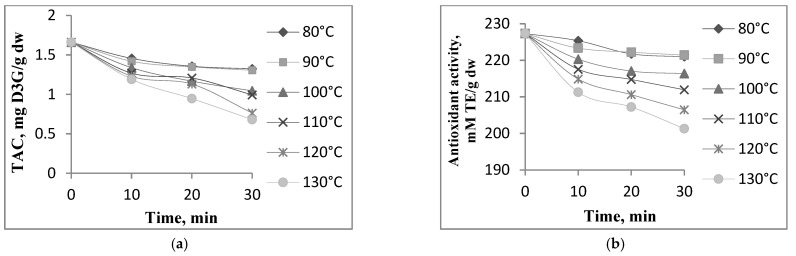
Degradation of total anthocyanin content—TAC (**a**) and antioxidant activity (**b**) in eggplant peel extract treated at different temperatures; D3G = delphinidin 3-*O*-glucoside; TE = Trolox Equivalent; dw—dry weight.

Anthocyanins are responsible for the purple color of the eggplant peels, which are highly susceptible to degradation during processing, resulting in color changes. To evaluate the degradation behavior of the phenolics from eggplant peel extract, a thermal treatment was performed at different temperature–time combinations. The combinations tested caused changes in TAC and antioxidant activity (data not shown, *p* < 0.05) except for the antioxidant activity at 90 °C (Figure 3a,b). The thermal processing techniques used in the industry usually have an impact on the structures of the phenolic compounds, influencing their bioavailability and bioactivities [18].

In our study at 80 °C, the TAC decreased by 20% after 30 min when compared with the untreated extract (Figure 3a). At 130 °C, the TAC decreased by 59% after 30 min. The antioxidant activity was less heat sensitive, with a reduction of 2.8% and 12% after 30 min of treatment at 80 and 130 °C, respectively (Figure 3b). In the whole temperature and time range studied, a similar trend was observed for TAC and antioxidant activity. Our results are in agreement with different studies. A sequential reduction in the phytochemicals and the antioxidant activity from a sour cherry extract was also reported by Oancea et al. [36] in the whole temperature range they studied. In contrast, Slavu et al. [37] reported no significant heat-induced changes in the total anthocyanin content from the purple maze extract in the temperature range of 80 to 110 °C.

#### 2.5.1. Kinetic Analysis

Kinetic models are often used to assess the influence of processing on the critical quality parameters of products [38]. For the eggplant peel extract, the kinetic parameters at constant pH = 2.18 and temperatures ranging from 80 to 130 °C for 0 to 30 min were studied by spectrophotometric analysis. The linear relation between the logarithm of TAC and antioxidant activity and time indicated that the degradation followed the first-order reaction kinetics. The kinetic parameters describing the heat-induced changes are the degradation rate (1/min) and degradation energy of activation (Ea) and are presented in Table 4.

The k values for TAC increased with increasing temperature, suggesting a high thermal sensibility at higher temperatures. In the antioxidant activity case, the k values were lower when compared to the TAC, but also increasing with the temperature from 80 to 130 °C. Our results are in agreement with other authors who have also reported the first-order kinetic model for the degradation of phenolics. Turturica et al. [39] reported an accelerated degradation effect on the TAC from plum extracts determined by the temperature increase in the 70–110 °C range. Qiu et al. [40] noticed that k values increased with the temperature in the 50–80 °C range after the thermal treatment of purple potato extract. Similar to our study, Oancea et al. [41] noticed that the k values for the antioxidant activity of elderberry extract were lower when compared with the TAC thermal degradation, but also increasing with the temperature.

The t1/2 values of TAC and antioxidant activity of eggplant peel extract decreased with increasing the heating temperature (Table 4) and were determined from the k values. Therefore, the higher the k value, the shorter the t1/2 value. The anthocyanins were the most susceptible to thermal degradation. This may be due to the anthocyanin oxidation or cleavage of covalent bonds during heat treatment. The antioxidant activity instead had the highest half-life, regardless of the heating temperature. Our results for the t1/2 at 80 °C are higher than those reported by Turturica et al. [39] at the same temperature for a plum extract regarding the TAC and antioxidant activity. Slavu et al. [37] also reported lower values of t1/2 than ours for the TAC after thermally treating a purple maze extract at 130 °C. On the contrary, Oancea et al. [36] obtained higher t1/2 values than ours for a thermally treated sour cherry extract at 130 °C, regarding the TAC and antioxidant activity. All the authors reported a higher susceptibility of anthocyanins to thermal degradation than other compounds.

The activation energy helps to characterize the temperature dependence of the parameters. This parameter allows a deeper understanding of the thermal degradation kinetics used to describe the required energy to reach the active state of a reaction. The degradation constants fitted to the Arrhenius equation, and the resulting Ea is shown in Table 4. Higher Ea values suggest that a smaller temperature change is required to faster degrade the compounds. Our results for the Ea of the TAC are similar to those reported by Turturica et al. [39] for a plum extract, but lower than those reported by Oancea et al. [36] for a sour cherry extract and by Slavu et al. [37] for a purple maze extract.

#### 2.5.2. Thermodynamic Parameters

Table 5 provides the results for the activation enthalpy (ΔH), the Gibbs free energy of inactivation (ΔG), and the activation entropy (ΔS) for the degradation of the anthocyanins and antioxidant activity from the eggplant peel extract at each temperature tested.

The activation enthalpy describes the endothermic state between the activated complex and the reactant. It is a measure of the energy barrier that must be overcome by the reacting molecules, related to the strength of the bonds that are broken and remade during the transition state [42]. The ΔH values calculated at different temperatures (Table 5) were similar. Considering that the ΔH values are positive, we can assume that the bioactive degradation was an endothermic reaction.

The Gibbs free energy of inactivation represents the difference between the activated state and the reactants state and describes its equilibrium and spontaneity [42]. The ΔG values calculated (Table 5) were positive for all temperatures tested, demonstrating that the bioactive degradation reaction is not spontaneous.

The activation entropy describes the change in the disorder of a system’s molecules [42]. The ΔS values calculated for each temperature (Table 5) were negative. These values demonstrate that the molecules from the transition state are more organized than at the beginning of the reaction, and the thermal degradation process is irreversible. Thus, the formation of the activated complex is associated with a decrease in entropy values.

## 3. Materials and Methods

### 3.1. Chemicals

EtOH from Titolchimica (Pontecchio Polesine, Italy), HCl, HPLC purity methanol (MeOH), Folin-Ciocalteau reagent (FC), potassium chloride solution (KCl), sodium acetate solution (CH_3_COONa), 2,2-diphenyl-1-picrylhydrazyl (DPPH) reagent, lipoxidase from Glycine max (soybean) type I-B, 50,000 units/mg protein, pancreatin lipase (111.5 units/mg protein), α-amylase from porcine pancreas (type I-A, 700–1400 units/mg protein), sodium phosphate buffer solution (PBS), linoleic acid (≥99%), p-nitrophenyl palmitate, Arabic gum, Triton X-100, starch solution, dinitrosalicylic acid (DNS), quercetin ≥95% (HPLC), orlistat ≥ 98%, and 1,1-Dimethylbiguanide hydrochloride (metformin hydrochloride) were purchased from Sigma Aldrich (Steinheim am Albuch, Germany). The standards used for the HPLC analysis, delphinidin 3-*O*-glucoside, delphinidin 3-*O*-rutinoside, and cyanidin 3-*O*-rutinoside, were purchased from Sigma Aldrich (Steinheim am Albuch, Germany) as primary reference standards.

### 3.2. Sample Preparation

The fresh fruits of an autochthonous eggplant (*Solanum melongena* L.) variety of a dark purple color with an elongated shape, named “Dragaica”, were purchased from a local market in Galați, Romania, in 2019. The fruits were washed, and the peels were removed with a knife in strips of uniform thickness. Subsequently, the peels were washed with ultrapure water, dried with paper towels, and frozen. The drying process was carried out with a CHRIST Alpha 1-4 LD plus equipment (Osterode am Harz, Germany), at −42 °C, under a pressure of 0.10 mBar, for 48 h until a 90% dw. The freeze-dried peels were grounded with MC 12 equipment, produced by Stephan (Germany), and stored in a plastic container with a lid in darkness at room temperature. The variability of the environmental conditions, such as the relative humidity and temperature, was set at the maximum possible. The dried material was analyzed in a maximum of 2 days.

### 3.3. The SLE Method

SLE was performed as a conventional method to extract the polyphenols from the eggplant peels [24]. The EtOH was used as a solvent in 70% and 96% concentrations. Based on previous preliminary results (data not shown), the 1N HCl was chosen to acidify the solvent in a 4:1 ratio of solvent to acid. The extractions took place under stirring at 25 and 50 °C, for 30 to 120 min, on an orbital shaker (SI-300R Medline Scientific, Chalgrove, UK) at 150 rpm. The solid-to-liquid ratio was set at 1:15 of plant material to solvent based on previous preliminary results (data not shown). Afterward, the samples were centrifuged using a Hettich Universal 320R, Germany, for 10 min at 14,000 rpm and 4 °C, and the supernatant was phytochemically analyzed.

### 3.4. The UAE Method

UAE was carried out as the Dranca and Oroian [25] method stated but with slight modifications. The method used EtOH 70% and 96% acidified with 1N HCl (4:1 ratio) at 25 and 50 °C, for 15 to 45 min, in a Smart sonic cleaner ultrasonic bath (MRC. Ltd., Holon, Israel). Previously, the samples were centrifuged for 10 min at 14,000 rpm and 4 °C, and the supernatant was further analyzed.

### 3.5. Characterization of Extracts

The TACs were quantified using the pH-differential method [43], and the results were expressed in milligrams of delphinidin 3-*O*-glucoside per gram of dry weight of peels (mg D3G/g dw) ±SD. The experiment was performed in triplicate, using a 0.025 M KCl buffer at pH = 1.0 and 0.4 M CH_3_COONa buffer at pH = 4.5. The absorbances were measured at 520 and 700 nm with a double-beam UV-VIS spectrophotometer with data analysis software (JENWAY, UK). The TAC was assessed according to Equation (1).

TAC (mg D3G/g dw) = (A × MW × DF)/ε × L(1)

where, A = (A520 nm − A700 nm) pH 1.0 − (A520 nm − A700 nm) 4.5(2)

MW—molecular weight of delphinidin 3-*O*-glucoside (465 g/mol); DF—dilution factor; ε—the molar absorptivity of delphinidin 3-*O*-glucoside (29,000 L × mol^−1^ × cm^−1^); L—the optical path (1 cm).

### 3.6. In Vitro Antioxidant Activity

To measure the in vitro antioxidant activity, DPPH (2,2-diphenyl-1-picrylhydrazyl) free radical was used, and the results were expressed as millimoles Trolox Equivalent per gram of dry weight of the peels (mM TE/g dw) ±SD [39]. A volume of 100 μL of the extract was mixed with 3.9 mL of DPPH stock solution. Afterward, the mixture was kept in darkness at room temperature for 30 min. The absorbance was recorded at λ = 515 nm against the blank with a double-beam UV-VIS spectrophotometer with data analysis software (Jenway, Staffordshire, UK). The results were calculated based on the Trolox calibration curve (n = 3), using Equation (3).

Antioxidant activity (mM TE/g dw) = (CE × V × d)/m(3)

CE—concentration in the calibration curve; V—stock volume; d—sample dilution; m—sample mass taken into analysis.

### 3.7. HPLC Analysis of the Anthocyanins

The anthocyanin chromatographic separation and identification were achieved by the method described by Condurache et al. [44]. A Thermo Finnigan Surveyor HPLC system coupled with a DAD detector and controlled by the Xcalibur software (Finnigan Surveyor LC, Thermo Scientific, USA) was used. In order to separate and identify the anthocyanins from the eggplant peels, a Synergi 4u Fusion-RP 80A (150 × 4.6 mm, 4 μm) column was used at an oven temperature of 25 °C, at 520 and 280 nm wavelengths. The samples were pre-filtered using a C18 Sep-Pack cartridge, in order to remove any unwanted compounds other than the targeted ones. Afterward, they were filtered through 0.22 μm syringe filters (Bio Basic Canada Inc., Markham ON, Canada). The solvents used for the elution step were 10% formic acid (A) and 100% methanol (B) under the following gradient conditions: 0–20 min, 9–35% (A); 20–30 min, 35% (A); 30–40 min, 35–50% (A); and 40–55 min, 50–90% (A). The injection volume was 10 μL at a flow rate of 1.0 mL/min. The identification and separation of the compounds were achieved based on their standard curves (delphinidin 3-*O*-glucoside, delphinidin 3-*O*-rutinoside, and cyanidin 3-*O*-rutinoside), retention times, and the data reported in the literature by Ferarsa et al. [5] and Dranca and Oroian [25]. All the used standard compounds presented a purity of ≥95.0% and were bought from Sigma Aldrich (Germany).

Further, the extracts were concentrated until to dryness under reduced pressure at 40 °C (AVC 2-18, Christ, UK) and stored at 4 °C in Falcon tubes. The dried extracts were used for the following analysis in a maximum of 2 days.

### 3.8. In Vitro Enzymes Inhibition Capacity of Eggplant Peel Extracts

#### 3.8.1. In Vitro LOX Inhibition Assay

The bioassay for the LOX inhibition was performed according to Costamagna et al. [45], with minor modifications. Briefly, 50 μL of 1, 0.5, and 0.1 mg/mL of extract diluted in ultrapure water was added to 50 μL of LOX solution (1 mg/mL in 0.1 M PBS, pH = 9.0). After 5 min of incubation at room temperature, 50 μL of 0.05 mM linoleic acid dissolved in 0.1 M PBS (pH = 9.0) was added, and the mixture was incubated for 20 min at 37 °C. Further, the samples were diluted with 2 mL of 0.1 M PBS at pH = 9.0, and the absorbances were read at 234 nm in quartz cuvettes with a double-beam UV-VIS spectrophotometer with data analysis software (Jenway, UK). Quercetin was used as a standard inhibitor. The inhibitory effect of the extracts was expressed as the IC50 value and the percentage of enzyme activity inhibition. The IC50 value (µg/mL) represents the extract concentration at which 50% of the enzyme activity is inhibited and was determined graphically by plotting the logarithmic concentration versus the inhibition percentage. The enzyme activity inhibition was calculated using the following equation:

% inhibition = (A_0_ − A_s_)/A_0_ × 100 (4)

A_0_ is the absorbance of the control sample without extract; A_s_ is the absorbance of the sample with the extract.

#### 3.8.2. In Vitro Lipase Inhibition Assay

The lipase inhibitory effect of the eggplant peels extract was also estimated using the method described by Costamagna et al. [45], with minor modifications. A volume of 50 μL of 1, 0.5, and 0.1 mg/mL extract was added to 50 μL of pancreatin lipase solution (1 mg/mL in 0.1 M PBS, pH = 8.0). After 5 min of incubation at room temperature, 50 μL of the substrate obtained from 0.01 M p-nitrophenyl palmitate, Arabic gum, and Triton x-100 was added, and the mixture was incubated for 20 min at 37 °C. Further, the samples were diluted with 1 mL of 0.1 M PBS at pH = 8.0, and the absorbances were read at 400 nm with a double-beam UV-VIS spectrophotometer with data analysis software (Jenway, UK). Orlistat was used as the standard inhibitor. The inhibitory effect of the extracts was expressed as IC50 and as the percentage of enzyme activity inhibition, calculated using Equation (4).

#### 3.8.3. In Vitro α-Amylase Inhibition Assay

In order to assess the α-amylase inhibitory effect of the eggplant peel extract, 100 μL of 1, 0.5, and 0.1 mg/mL samples was added to 100 μL of α-amylase solution (1 mg/mL in 0.1 M PBS, pH 6.9). After 5 min of incubation at room temperature, 100 μL of soluble starch 1% solution dissolved in distilled water and previously boiled for 5 min was added to each tube and incubated for another 20 min at 37 °C. Finally, in each test tube, 200 μL of DNS reagent was added, followed by heating at 100 °C for 5 min in a thermostatic water bath (Digibath-2 BAD 4, Raypa Trade, Barcelona, Spain). Further, the samples were diluted with 2 mL of distilled water, and the absorbance was read at 540 nm with a double-beam UV-VIS spectrophotometer with data analysis software (Jenway, UK). Metformin hydrochloride was used as the standard inhibitor. The inhibitory effect of the extracts was expressed as IC50 and as the percentage of enzyme activity inhibition, calculated using Equation (4) [45].

### 3.9. TAC and Antioxidant Activity Thermal Degradation

#### 3.9.1. Heat Treatment

For the kinetic studies, glass tubes were filled with 2 mL of the extract solution (10 mg/mL in ultrapure water, pH = 2.18) and heated in the 80 to 130 °C temperature range for 10 to 30 min, using a digital heating block (Stuart, UK). The parameters were chosen considering the pasteurization and sterilization temperatures and times. After the thermal treatment, the tubes were cooled in a mixture of ice and water to prevent further degradation. Finally, the TAC and antioxidant activity of the thermally treated extract were analyzed using the spectrophotometric methods described above.

#### 3.9.2. Mathematical Models and Kinetic Analysis

The degradation kinetics of TAC and antioxidant activity was described by fitting the experimental data to the first-order kinetic model (Equation (5)):

C/C0 = e − kt(5)

where C0 and C are the TAC or antioxidant activity before and after thermal treatment (mg/g dw), respectively; t is the heating time (min); and k is the degradation rate constant at T temperature.

Starting from Equation (5), the following kinetic parameters were calculated: the degradation rate constant (k), the half-life of degradation (t1/2), and the activation energies (Ea), as described by Slavu (Ursu) et al. [37].

#### 3.9.3. Thermodynamic Parameters

The activation enthalpy (ΔH), the Gibbs free energy of inactivation (ΔG), and the activation entropy (ΔS) at each thermal treatment temperature were calculated as suggested by Radu (Lupoae) et al. [46].

### 3.10. Statistical Analysis of Data

The statistical analysis of data was performed using the IBM SPSS 2020 Statistics Software. The differences between the samples were assessed by the Tukey test with the One-way ANOVA method for the data that followed the normal distribution and equal variances conditions. For the other ones, the Kruskal–Wallis and Dunn’s nonparametric tests were used. All the experiments were expressed as average values with a standard deviation of triplicates.

## 4. Conclusions

Two different methods (SLE and UAE) were evaluated in terms of the best extraction yield and high quality of extracts. Two different concentrations of EtOH were chosen, at different extraction times and temperatures. Both extraction methods presented a high anthocyanin content at the same EtOH concentration (96%) and time (30 min). The chromatographic profile revealed the presence of five compounds: delphinidin 3-*O*-rutinoside-5-glucoside, delphinidin 3-*O*-glucoside, delphinidin 3-*O*-rutinoside, cyanidin 3-*O*-rutinoside, and petunidin 3-*O*-rutinoside. The anthocyanin with the highest concentration from each extract was delphinidin 3-*O*-rutinoside, with a concentration 3.5 times higher in UAE extract than in SLE extract. Both extracts presented moderate in vitro inhibitory activity in LOX, lipase, and α-amylase. This fact suggests that the phenolics from the eggplant peel extracts might be involved in reducing the occurrence of various disorders when there is a high bioavailability in the human body. However, UAE inhibited the enzymes in a significantly higher percentage than SLE (*p* < 0.05). Thereby, UAE is preferable to SLE due to the lower temperature used, higher delphinidin 3-*O*-rutinoside content, and enzymes inhibitory activity. The degradation data of eggplant peel anthocyanins and antioxidant activity follow the first-order reaction kinetics, allowing an accurate prediction of the thermal degradation kinetic parameters. The kinetic and thermodynamic parameters showed that the temperature increase had to accelerate the degradation effect, demonstrating a higher temperature dependence of anthocyanins and antioxidant activity. This information is useful for optimizing the industrial processing conditions in order to minimize losses in phenolic compounds. The eggplant peel extracts have an outstanding potential to be used as paint ingredients, curing agents, and textile dyes, etc., though stability is among the main challenges that must be overcome. To overcome the limitations of the use of eggplant peel anthocyanins, alternatives, such as encapsulation, can allow loss minimization and increase the compounds’ half-life. In this regard, more research will be further addressed.

## Figures and Tables

**Table 1:** The total anthocyanin content (TAC) and antioxidant activity of the extracts obtained with solid–liquid extraction (SLE) and ultrasound-assisted extraction (UAE) methods by varying the extraction parameters.

**(a) TAC, mg D3G/g dw**					
**Samples**	**t, min**	**EtOH 70%**		**EtOH 96%**	
		**25 °C**	**50 °C**	**25 °C**	**50 °C**
SLE	30	0.84 ± 0.05 ^a*1◌^	1.01 ± 0.08 ^a#1◌^	0.93 ± 0.06 ^a*2◌^	1.11 ± 0.08 ^a#1◌^
	60	0.84 ± 0.07 ^a*1^	0.96 ± 0.09 ^a#1^	0.92 ± 0.09 ^a*1^	1.13 ± 0.10 ^a#2^
	120	0.87 ± 0.03 ^a*1^	1.02 ± 0.09 ^a#1^	1.01 ± 0.10 ^a*2^	1.06 ± 0.06 ^a#1^
UAE	15	0.91 ± 0.08 ^A*1^	0.87 ± 0.05 ^A*1^	0.98 ± 0.09 ^A*1^	0.79 ± 0.07 ^A#2^^●^
	30	0.90 ± 0.08 ^A*1^^◌^	0.93 ± 0.09 ^A*1^^◌^	1.04 ± 0.10 ^A*2^^◌^	0.75 ± 0.07 ^A#2^
	45	0.94 ± 0.09 ^A*1^	0.90 ± 0.08 ^A*1^	0.97 ± 0.07 ^A*1^	0.95 ± 0.09 ^B*1^
**(b) Antioxidant Activity, mM TE/g dw**					
**Samples**	**t, min**	**EtOH 70%**		**EtOH 96%**	
		**25 °C**	**50 °C**	**25 °C**	**50 °C**
SLE	30	25.34 ± 1.88 ^a*1◌^	39.36 ± 2.87 ^a#1◌^	31.89 ± 3.02 ^a*2◌^	34.92 ± 3.41 ^a*2◌^
	60	28.80 ± 2.42 ^b*1^	39.85 ± 3.19 ^a#1^	30.96 ± 2.81 ^a*1^	38.97 ± 1.75 ^b#1^
	120	27.67 ± 1.35 ^ab*1^	38.17 ± 3.47 ^a#^	29.75 ± 2.04 ^a*1^	32.61 ± 2.5^a*2^
UAE	15	34.31 ± 0.42 ^A*1^	32.10 ± 1.99 ^A*1^	22.46 ± 1.01 ^A*2^	32.64 ± 2.44 ^A#1^
	30	32.74 ± 0.38 ^B*1◌^	33.54 ± 0.63 ^A#1◌^	20.64 ± 1.93 ^A*2◌^	32.86 ± 1.76 ^A#1●^
	45	32.59 ± 0.76 ^B*1^	32.10 ± 1.99 ^A*1^	21.69 ± 0.99 ^A*2^	32.65 ± 1.34 ^A#1^

The influence of extraction time was highlighted using lowercase and uppercase letters for SLE and UAE, respectively, in a column. The influence of extraction temperature and solvent concentration was highlighted using symbols (^*^, ^#^) and digits, respectively, in lines. The differences between the extraction methods were highlighted using symbols (◌, ●) in a column. Values that share a letter/symbol/digit are not significantly different (*p* > 0.05). TAC = total anthocyanin content; EtOH = ethanol; t = time; D3G = delphinidin 3-*O*-glucoside; TE = Trolox Equivalent; dw = dry weight.

**Table 2:** The enzyme inhibition percentage of SLE and UAE selected extracts and positive controls in LOX, lipase, and α-amylase enzymes at different concentrations.

Sample Concentration, mg/mL		LOX Inhibition, %	Lipase Inhibition, %	α-Amylase Inhibition, %
SLE	1	50.87 ± 0.84 ^a^	25.35 ± 0.35 ^a^	58.58 ± 2.09 ^a^
	0.5	49.52 ± 0.35 ^d^	23.31 ± 1.22 ^c^	56.73 ± 1.36 ^bc^
	0.1	46.59 ± 1.81 ^g^	22.29 ± 093 ^e^	47.51 ± 1.22 ^d^
UAE	1	53.64 ± 0.45 ^b^	26.25 ± 1.04 ^a^	58.02 ± 1.01 ^a^
	0.5	52.95 ± 0.75 ^e^	25.34 ± 0.68 ^c^	54.26 ± 1.57 ^c^
	0.1	49.13 ± 3.38 ^g^	24.42 ± 1.72 ^e^	48.50 ± 2.89 ^d^
Quercetin	1	84.98 ± 3.28 ^c^	-	-
	0.5	79.37 ± 0.60 ^f^	-	-
	0.1	77.32 ± 1.20 ^h^	-	-
Orlistat	1	-	34.70 ± 2.59 ^b^	-
	0.5	-	32.19 ± 0.68 ^d^	-
	0.1	-	30.82 ± 0.68 ^f^	-
Metformin hydrochloride	1	-	-	60.68 ± 0.83 ^a^
	0.5	-	-	58.91 ± 1.57 ^b^
	0.1	-	-	56.36 ± 1.01 ^e^

Values from a column for a similar concentration that share a letter are not significantly different (*p* > 0.05). Measurements are expressed as mean ± SD of triplicates. SLE—solid/liquid conventional extraction; UAE—ultrasound-assisted extraction; LOX—lipoxygenase.

**Table 3:** The enzyme inhibition results (IC50 values; μg/mL) of SLE and UAE selected extracts in LOX, lipase, and α-amylase enzymes.

Sample	IC50 (μg/mL)		
	LOX	Lipase	α-amylase
SLE	2.80 ± 0.68 ^a^	3.31 ± 0.24 ^a^	10.72 ± 1.06 ^a^
UAE	2.83 ± 0.44 ^a^	2.59 ± 0.24 ^a^	7.27 ± 0.23 ^b^
Quercetin	7.81 ± 0.66 ^b^	-	-
Orlistat	-	1.23 ± 0.09 ^b^	-
Metformin hydrochloride	-	-	4.31 ± 0.48 ^c^
SLE	2.80 ± 0.68 ^a^	3.31 ± 0.24 ^a^	10.72 ± 1.06 ^a^

Values from a column that share a letter are not significantly different (*p* > 0.05). Measurements are expressed as mean ± SD of triplicates. SLE—solid/liquid conventional extract; UAE—ultrasound-assisted extract; LOX-lipoxygenase.

**Table 4:** Estimated kinetic parameters of bioactives in eggplant peel extract.

Compounds	T, °C	K × 10^2^, min^−1^	R^2^	t1/2, min	Ea, kJ/mol
TAC	80	0.74 ± 0.001	0.87	93.67 ±1.05	34.63 ± 3.59
	90	0.79 ± 0.004	0.90	87.74 ± 0.78	
	100	1.53 ± 0.002	0.97	45.30 ± 0.44	
	110	1.59 ± 0.001	0.94	43.59 ± 4.84	
	120	2.42 ± 0.003	0.94	28.64 ± 0.33	
	130	2.91 ± 0.01	0.99	23.82 ± 0.71	
Antioxidant activity	80	0.10 ± 0.001	0.95	693.14 ± 4.87	37.24 ± 3.24
	90	0.08 ± 0.001	0.86	866.43 ± 4.76	
	100	0.16 ± 0.02	0.86	433.21 ± 5.21	
	110	0.22 ± 0.001	0.89	315.06 ± 3.50	
	120	0.31 ± 0.04	0.92	223.59 ± 1.51	
	130	0.38 ± 0.001	0.91	182.40 ± 1.46	

TAC—total anthocyanin content; k—the degradation rate constant; t1/2—the half-life of degradation; Ea—the activation energies; R^2^—determination coefficient; T—temperature.

**Table 5:** Thermodynamic parameters obtained for phytochemical degradation in eggplant peel extract.

Compounds	T, °C	ΔH, kJ/mol	ΔG, kJ/mol	ΔS, J·mol^−1^·K^−1^
TAC	80	31.69 ± 1.03	113.36 ± 9.56	−231.34 ± 11.05
	90	31.61 ± 0.40	116.46 ± 11.61	−233.74 ± 18.80
	100	31.53 ± 1.20	117.70 ± 10.98	−231.08 ± 15.21
	110	31.44 ± 1.10	120.82 ± 9.03	−233.35 ± 11.55
	120	31.36 ± 3.01	122.68 ± 9.43	−232.37 ± 15.13
	130	31.28 ± 0.10	125.27 ± 11.26	−233.23 ± 17.01
Antioxidant activity	80	34.30 ± 1.72	119.23 ± 12.68	−240.60 ± 14.67
	90	34.22 ± 2.01	123.37 ± 15.49	−245.59 ± 14.76
	100	34.13 ± 1.99	124.70 ± 10.71	−242.80 ± 15.82
	110	34.05 ± 1.53	127.12 ± 11.35	−242.98 ± 13.35
	120	33.97 ± 1.67	129.40 ± 12.99	−242.82 ± 11.01
	130	33.89 ± 0.91	132.09 ± 11.47	−243.69 ± 11.89

TAC—total anthocyanin content; ΔH—activation enthalpy; ΔG—Gibbs inactivation free energy; ΔS—activation entropy; T—temperature.

## Data Availability

The data that support the findings of this study are available from the corresponding author, [G.R.], upon reasonable request.
